# Cooperative assembly of filopodia by the formin FMNL2 and I-BAR domain protein IRTKS

**DOI:** 10.1016/j.jbc.2022.102512

**Published:** 2022-09-19

**Authors:** Sarah Fox, Amanda Tran, Laura Trinkle-Mulcahy, John W. Copeland

**Affiliations:** Department of Cellular and Molecular Medicine, Faculty of Medicine, University of Ottawa, Ottawa, Ontario, Canada

**Keywords:** formin, actin, cell motility, filopodia, membrane, melanoma, co-IP, coimmunoprecipitation, FH, formin homology, FMNL, formin-like, HEK293T, human embryonic kidney 293T cell line, I-BAR, Inverse-Bin-Amphiphysin-Rvs, PEI, polyethyleneimine, RRID, Research Resource Identifier

## Abstract

Filopodia are long finger-like actin-based structures that project out from the plasma membrane as cells navigate and explore their extracellular environment. The initiation of filopodia formation requires release of tension at the plasma membrane followed by the coordinated assembly of long unbranched actin filaments. Filopodia growth is maintained by a tip complex that promotes actin polymerization and protects the growing barbed ends of the actin fibers from capping proteins. Filopodia growth also depends on additional F-actin bundling proteins to stiffen the actin filaments as well as extension of the membrane sheath projecting from the cell periphery. These activities can be provided by a number of actin-binding and membrane-binding proteins including formins such as formin-like 2 (FMNL2) and FMNL3, and Inverse-Bin-Amphiphysin-Rvs (I-BAR) proteins such as IRTKS and IRSp53, but the specific requirement for these proteins in filopodia assembly is not clear. We report here that IRTKS and IRSp53 are FMNL2-binding proteins. Coexpression of FMNL2 with either I-BAR protein promotes cooperative filopodia assembly. We find IRTKS, but not IRSp53, is required for FMNL2-induced filopodia assembly, and FMNL2 and IRTKS are mutually dependent cofactors in this process. Our results suggest that the primary function for FMNL2 during filopodia assembly is binding to the plasma membrane and that regulation of actin dynamics by its formin homology 2 domain is secondary. From these results, we conclude that FMNL2 initiates filopodia assembly *via* an unexpected novel mechanism, by bending the plasma membrane to recruit IRTKS and thereby nucleate filopodia assembly.

Filopodia are dynamic structures formed from bundles of long unbranched actin filaments that protrude from the plasma membrane. During cell migration, filopodia probe the extracellular environment for guidance cues and establish cell–substrate attachments to facilitate motility (1, 2, 3). Filopodia formation is dependent upon a specific series of membrane and cytoskeletal remodeling activities that begin by first relieving tension at the plasma membrane to permit filopodia growth. This is followed by assembly of a filopodia tip complex that promotes F-actin polymerization and uncapping of the barbed end of the actin filament. As the filopodia grows, the actin filaments are bundled to promote stiffness and provide force as they push against the membrane, which is also extended and tubulated by additional membrane bending proteins (1, 4, 5, 6). Composition of the tip complex is cell type dependent and may include formins, Ena/VASP, and Inverse-Bin-Amphiphysin-Rvs (I-BAR) proteins (3), and multiple factors are thought to be able to provide the individual activities required for filopodia growth (7).

Formin-like 2 (FMNL2) and FMNL3 are formin proteins associated with filopodia formation in multiple cell types (8, 9, 10, 11, 12, 13). Overexpression of FMNL2 and FMNL3 is sufficient to induce filopodia assembly (8, 14, 15), and inhibition of Arp2/3 activity induces abundant formation of FMNL2- and FMNL3-dependent filopodia. Like other formins, FMNL2 and FMNL3 regulate actin polymerization through two conserved functional domains formin homology 1 (FH1) and FH2 and act as “leaky” cappers at the barbed end of the actin filament. The FH2 domains of both proteins, in association with a C-terminal WH2-like motif, are also able to bind and bundle F-actin (16, 17, 18). Despite the homology shared between FMNL2 and its paralog FMNL3, they display distinct localizations within filopodia (3). Both FMNL2 and FMNL3 undergo N-myristoylation at glycine residue 2, which targets the proteins to the plasma membrane (19). Although it is not clear if this modification is required for their effects on actin cytoskeletal dynamics (20, 21), we noted previously that disruption of FMNL2 N-myristoylation blocks its ability to induce filopodia assembly (14). Based on its crystal structure, the FMNL2 N terminus is predicted to induce negative membrane bending when docked at the plasma membrane, and it was proposed that this might be sufficient to nucleate filopodia formation (12).

I-BAR (22, 23, 24, 25) proteins are membrane-binding proteins associated with filopodia assembly. The I-BAR domain homodimerizes to form a convex membrane-binding surface that binds preferentially to membranes enriched for PI(4,5)P_2_. Binding of the dimerized I-BAR domain to the plasma membrane is thought to both sense as well as induce negative membrane bending and is sufficient to induce membrane tubulation and formation of filopodia-like projections (26). Indeed, I-BAR-induced membrane bending is predicted to initiate filopodia assembly through the release of membrane tension at the leading edge of the cell (6). IRTKS (aka BAIAP2L1) is an I-BAR protein required for filopodia assembly downstream of the small GTPase Rif (27) and is also associated with microvilli formation in epithelial cells (28, 29). Similarly, IRSp53 (aka BAIAP2) cooperates with a variety of actin remodeling proteins to mediate filopodia assembly downstream of Cdc42. In addition to the N-terminal I-BAR domain, both proteins possess an SH3 domain that plays an autoregulatory role in governing I-BAR function (30). IRSp53 has an additional regulatory CRIB motif (25), and IRTKS possesses a C-terminal WH2-like actin-binding motif (30).

To shed further light on the role of FMNL2 in filopodia assembly, we used BioID to generate an FMNL2 interactome. From this screen, we identified IRTKS and IRSp53 as novel FMNL2-binding proteins. We show here that both IRTKS and IRSp53 cooperate with FMNL2 in filopodia assembly in human melanoma cells and that IRTKS and FMNL2 are mutually dependent cofactors in this process. Surprisingly, coexpression of IRTKS rescues filopodia assembly downstream of an FMNL2 mutant that is disabled in its ability to bind actin but does not rescue an FMNL2 mutant that cannot be myristoylated. Our results support a novel model for filopodia assembly, in which FMNL2-induced membrane bending initiates filopodia formation by recruiting IRTKS to the plasma membrane.

## Results

**Figure 1:**
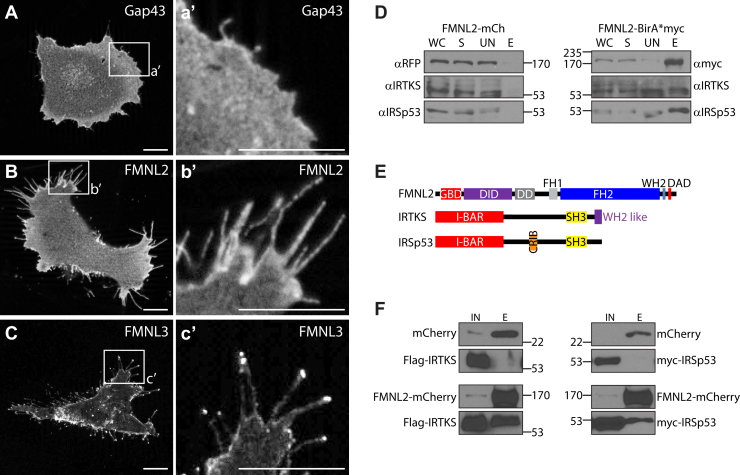
**FMNL2 localizes along the length of filopodia.***A*, *a'*, expression of the plasma membrane marker Gap43-mCherry in A2058 human melanoma cells does not induce filopodia assembly. *B*, transient expression of FMNL2-mCherry induces extensive filopodia formation in A2058 cells. FMNL2-mCherry is recruited to the plasma membrane and (*b'*) localizes along the length of the filopodia without concentrating at the filopodia tip. *C*, transient expression of FMNL3-mCherry in A2058 cells induces filopodia assembly and (*c'*) FMNL3 accumulates at the tips of filopodia. The scale bar represents 10 μm. *D*, FMNL2-BirA∗ expression in HEK293T/17 cells induces biotinylation of endogenous IRSp53 and IRTKS. Endogenous IRTSp53 and IRTKS are not biotinylated in cells expressing FMNL2-mCherry. HEK293T/17 cells transiently expressing FMNL2-mCherry or FMNL2-BirA∗ were lysed following treatment with exogenous biotin. Total biotinylated proteins were isolated from each sample using streptavidin-agarose beads. The eluted proteins were immunoblotted using the indicated antibodies. FMNL2-BirA was detected by immunoblotting for its myc epitope tag. *E*, schematic of FMNL2, IRSp53, and IRTKS protein structure. FMNL2 contains the regulatory N-terminal GTPase-binding (GBD) and DAD-interacting (DID) domains, the N-terminal dimerization domain (DD), proline-rich formin homology 1 (FH1), the functional FH2 domain, a wasp homology 2 (WH2) motif, and the diaphanous autoregulatory domain (DAD). IRSp53 and IRTKS contain a related membrane-binding I-BAR domain and SH3 domain but differ by the presence of a Cdc42 and Rac interactive binding (CRIB) domain in IRSp53 and a C-terminal WH2-like domain in IRTKS. *F*, FMNL2-mCherry, but not mCherry alone, is able to coimmunoprecipitate IRTKS (*left panels*) and IRSp53 (*right panels*). mCherry is efficiently immunoprecipitated using RFP-trap agarose but fails to co-IP coexpressed FLAG-tagged IRSp53 or myc-tagged IRTKS. FMNL2-mCherry co-IPs coexpressed IRTKS and IRSp53. E, eluate fraction; FMNL, formin-like; HEK293T, human embryonic kidney 293T cell line; IN, soluble lysate input; S, soluble lysate fraction; UN, unbound fraction; WC, whole cell lysate.

FMNL2 is required for melanoma cell migration, where it generates protrusive force to push forward the lamellipodia (20, 21). FMNL2 is also associated with filopodia assembly (11, 12, 31), and the most prominent phenotype when FMNL2 is overexpressed in many cell types is excessive filopodia formation, an effect that is dependent on the integrity of its N-terminal myristoylation sequence (14, 19). We noted that when transiently expressed in A2058 melanoma cells, FMNL2-mCherry displays a distinct subcellular localization along the length of the filopodia without an obvious concentration at the filopodia tip (Fig. 1, *A* and *B*). This is in clear contrast to its paralog FMNL3, which obviously accumulates at the tips of filopodia when expressed in these cells (Fig. 1*C*). Although both proteins are N-myristoylated, this differential localization suggests that FMNL2 and FMNL3 induce filopodia assembly *via* distinct mechanisms.

In an effort to gain further insight into the distinct role of FMNL2 in filopodia assembly, we used BioID (32) to identify potential FMNL2-interacting proteins. An FMNL2-BirA∗ fusion derivative was generated, and a BioID screen was carried out in human embryonic kidney 293T (HEK293T) cells. From this screen, we identified two proteins of interest as potential FMNL2-binding partners: the I-BAR proteins IRTKS (aka BAIAP2L1) and IRSp53 (BAIAP2). Both proteins are known to participate in filopodia formation (25, 27, 30, 33, 34). To validate these proteins as *bona fide* FMNL2-interacting proteins, we first tested the ability of FMNL2-BirA∗ to biotinylate endogenous IRTKS and IRSp53 proteins (Fig. 1*D*). FMNL2-BirA∗ was expressed in HEK293T cells, lysates were prepared from the biotin-treated cells, and the biotinylated proteins were isolated using Streptavidin agarose beads. FMNL2-mCherry transfected cells were used as a control. The streptavidin-bound proteins were eluted, immunoblotted, and probed for the presence of endogenous IRTKS or IRSp53. The two I-BAR proteins were only detected in the FMNL2-BirA∗ lysate and not in the FMNL2-mCherry control eluate, consistent with a specific interaction between FMNL2 and these two targets. The interactions were directly confirmed by coimmunoprecipitation (co-IP). FMNL2-mCherry, or mCherry alone, was coexpressed with FLAG-tagged derivatives of either IRTKS or IRSp53. The cells were lysed, and FMNL2-mCherry, or the mCherry control, was immunoprecipitated using RFP-Trap. The input and eluate samples were immunoblotted, and the resultant blots probed with FLAG antibody coupled to horseradish peroxidase to detect the FLAG-tagged proteins. Both IRTKS and IRSp53 were detected in the FMNL2-mCherry, but not mCherry alone, eluate samples, confirming their specific interaction with FMNL2 (Fig. 1*F*).

**Figure 2:**
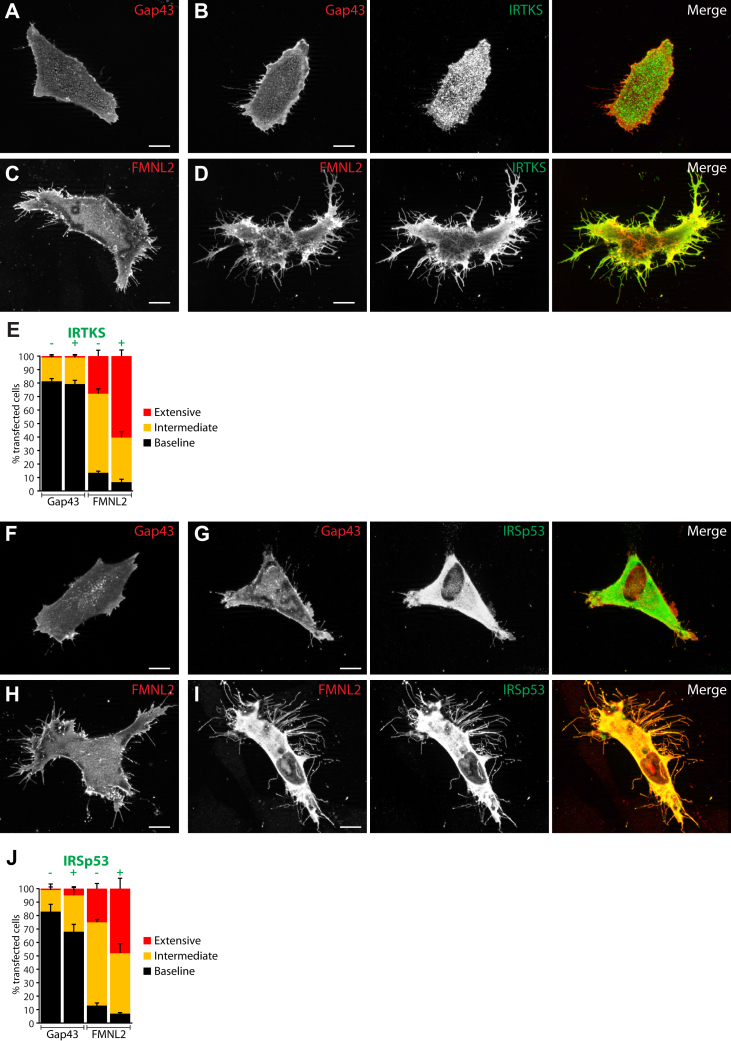
**FMNL2 and I-BAR protein coexpression induces extensive filopodia formation.***A*, Gap43-mCherry expression in A2058 cells does not induce filopodia formation. *B*, expression of FLAG-IRTKS induces assembly of moderate amounts of short filopodia. *C*, FMNL2-mCherry expression induces filopodia formation. *D*, coexpression of FLAG-IRTKS with FMNL2-mCherry induces extensive formation of dorsal and peripheral filopodia. The scale bar represents 10 μm. *E*, quantification of data shown in *A*–*D*. Percent of transfected cells with the indicated phenotypes. *Black bars*: baseline filopodia formation, *yellow bars*: intermediate filopodia formation, and *red bars*: extensive dorsal and peripheral filopodia (see Fig. S1 for cell morphology classification). N = 3, >100 cells/trial. Error bars represent SEM. *F*, as in (*A*), Gap43 mCherry expression in A2058 cells does not induce filopodia formation. *G*, expression of myc-IRSp53 has modest effects on the formation of peripheral filopodia. *H*, FMNL2-mCherry expression induces filopodia formation. *I*, coexpression of myc-IRSp53 with FMNL2-mCherry induces extensive formation of long peripheral filopodia. The scale bar represents 10 μm. *J*, quantification of data shown in *F*–*I*. Percent of transfected cells with the indicated phenotypes. *Black bars*: baseline filopodia formation, *yellow bars*: intermediate filopodia formation, and *red bars*: extensive dorsal or peripheral filopodia. N = 3, >100 cells/trial. Error bars represent SEM. Similar results were obtained in A375 human melanoma cells (Fig. S2). FMNL2, formin-like 2; I-BAR, Inverse-Bin-Amphiphysin-Rvs.

I-Bar proteins associate with phosphoinositide-rich membranes where docking of the convex I-BAR dimer generates negative bending in the target membrane (26, 33, 35). IRTKS and IRSp53 are thought to participate in filopodia assembly by relieving membrane tension at the cell periphery (33, 36). We coexpressed FMNL2-mCherry with either FLAG-tagged IRTKS or IRSp53 in A2058 melanoma cells and assessed their effects on filopodia formation by immunofluorescence (Figs. 2 and S1). On its own, FMNL2 induced an increase in the number of cells with amplified filopodia assembly in comparison to control cells expressing the plasma membrane marker Gap43-mCherry (37) (Fig. 2, *A* and *C*). On its own, epitope-tagged IRTKS localized to the cell periphery and to the tips of short filopodia but did not induce extensive filopodia formation (Fig. 2*B*). In contrast, coexpression of IRTKS with FMNL2 induced a striking phenotype with a significant increase in the number of cells with extensive dorsal and peripheral filopodia assembly and dorsal ruffling (Fig. 2, *D* and *E*). Likewise, coexpression of FMNL2 with IRSp53 also had cooperative effects on filopodia assembly. As with IRTKS, IRSp53 alone induced a moderate increase in filopodia assembly (Fig. 2, *F* and *G*), but coexpression of FMNL2 with IRSp53 greatly enhanced filopodia assembly (Fig. 2, *H*–*J*). Similar results were obtained with FMNL2 overexpression with IRTKS and IRSp53 in A375 human melanoma cells (Fig. S2).

**Figure 3:**
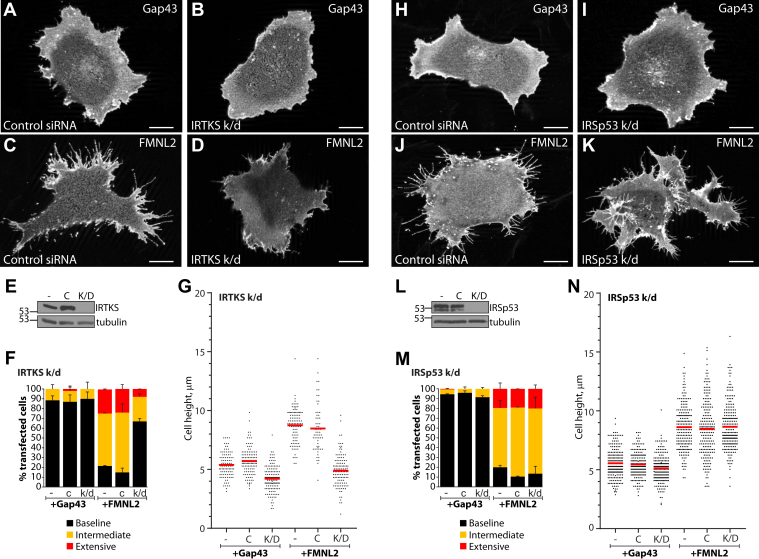
**FMNL2-induced filopodia assembly is IRTKS dependent.***A* and *B*, Gap43 mCherry expression does not affect filopodia formation in A2058 cells transfected with a control siRNA duplex or in IRTKS knockdown (k/d) cells. *C*, FMNL2-mCherry expression induces filopodia formation in control siRNA transfected cells. *D*, FMNL2-mCherry is still targeted to the plasma membrane in IRTKS-depleted cells, but filopodia formation is inhibited. The scale bar represents 10 μm. *E*, immunoblots confirming extent of IRTKS depletion in siRNA transfected cells. “-”: whole cell lysates from untransfected cells, C: whole cell lysates from control siRNA-transfected cells. K/D, whole cell lysates from cells transfected with siRNA targeting IRTKS. Tubulin was used as a loading control. *F*, quantification of data shown in *A*–*D*. Percent of transfected cells with the indicated phenotypes. *Black bars*: baseline filopodia formation, *yellow bars*: intermediate filopodia formation, and *red bars*: extensive dorsal and peripheral filopodia. N = 3, >100 cells/trial. Error bars represent SEM. *G*, FMNL2-mCherry expression in A2058 induces an increase in cell height that is inhibited by siRNA-mediated k/d of IRTKS expression. N = 3, >50 cells/trial, *red bars*: average height. Similar results were obtained in A375 cells and with a second siRNA duplex targeting IRTKS (Fig. S3). *H* and *I*, as in (*A*), Gap43-mCherry does not affect filopodia formation in A2058 cells transfected with a control siRNA duplex or in IRSp53 k/d cells. *J*, FMNL2-mCherry induces filopodia formation in control siRNA transfected cells. *K*, FMNL2-mCherry-induced filopodia formation is not inhibited in IRSp53-depleted cells. The scale bar represents 10 μm. *L*, immunoblots confirming extent of IRSp53 depletion in siRNA transfected cells. “-”: whole cell lysates from untransfected cells, C: whole cell lysates from control siRNA transfected cells, and K/D: whole cell lysates from cells transfected with siRNA targeting IRSp53. *M*, quantification of data shown in (*H*–*K*). Percent of transfected cells with the indicated phenotypes. *Black bars*: baseline filopodia formation, *yellow bars*: intermediate filopodia formation, and *red bars*: extensive dorsal and peripheral filopodia. N = 3, >100 cells/trial. Error bars represent SEM. *N*, siRNA-mediated k/d of IRSp53 expression does not inhibit FMNL2-mCherry-induced increases in cell height. N = 3, >50 cells/trial, *red bars*: average height. FMNL2, formin-like 2.

Together, these results suggest that I-BAR protein activity is a limiting factor in FMNL2-induced filopodia assembly. To test this idea, we used siRNA to knockdown IRTKS and IRSp53 expression in A2058 cells and assessed the effects on FMNL2-induced filopodia assembly and cell morphology (Fig. 3). IRTKS depletion did not have an obvious effect on cell morphology as visualized in cells transiently expressing the Gap43 membrane marker (Fig. 3, *A*, *B* and *F*). IRTKS knockdown strongly inhibited filopodia assembly in cells transiently expressing FMNL2. However, we noted that IRTKS depletion did not prevent FMNL2 from accumulating at the plasma membrane and cell periphery (Fig. 3, *C*, *D* and *F*). We also noted that in control cells, FMNL2 induced a striking increase in cell height because of the formation of abundant dorsal filopodia and dorsal protrusions and, as expected, IRTKS depletion also blocked this effect (Fig. 3*G*). Cell height in Gap43 control cells was also reduced by IRTKS knockdown (Fig. 3*G*). These results were confirmed in A375 cells as well as in A2058 cells using a second siRNA duplex targeting IRTKS (Fig. S3). In contrast, IRSp53 depletion did not affect FMNL2 activity. FMNL2 overexpression efficiently induced filopodia assembly in both control and IRSp53 knockdown cells (Fig. 3, *J*, *K* and *M*), and the FMNL2-induced increase in cell height was similarly unaffected (Fig. 3*N*). Thus, indicating that IRTKS, but not IRSp53, is required for FMNL2-induced filopodia assembly in these cells.

**Figure 4:**
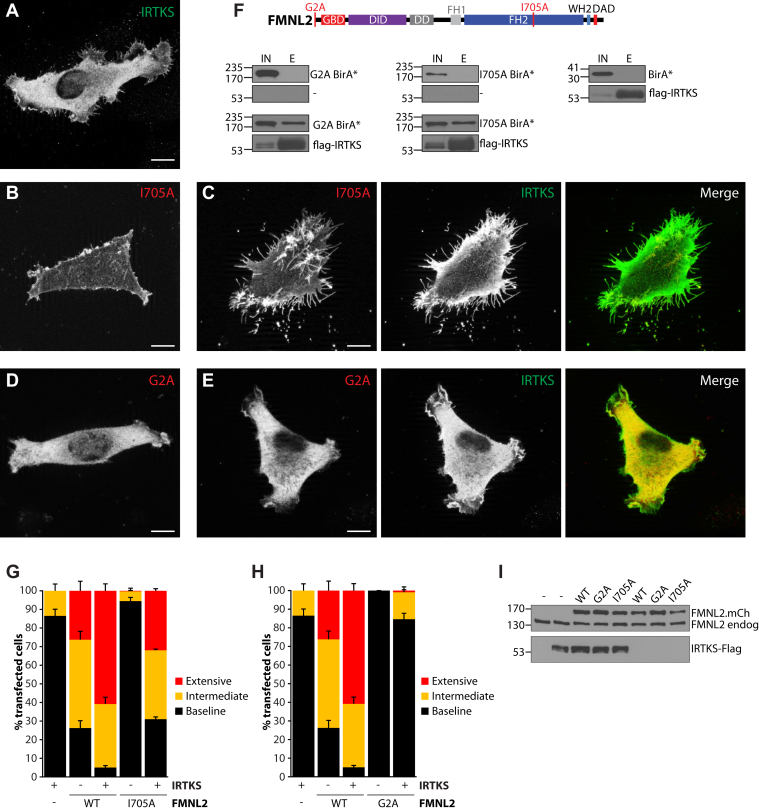
**IRTKS rescues filopodia assembly induced by an actin-defective mutant of FMNL2.***A*, transient expression of FLAG-tagged IRTKS induces moderate formation of short filopodia in A2058 cells. *B*, transiently expressed FMNL2.I705A-mCherry localizes to the plasma membrane but does not induce filopodia formation. *C*, coexpression of FLAG-IRTKS (*green*) with FMNL2.I705A-mCherry (*red*) induces extensive formation of dorsal and peripheral filopodia. *D*, transiently expressed FMNL2.G2A-mCherry (*red*) does not localize to the plasma membrane and does not induce filopodia formation. *E*, coexpression of FLAG-IRTKS (*green*) with FMNL2.G2A-mCherry (*red*) does not induce formation of dorsal and peripheral filopodia. The scale bar represents 10 μm. *F*, schematic indicating position of point mutations in FMNL2. *Top row*, FMNL2 derivatives tagged with BirA∗ are not pulled down by FLAG affinity beads alone. FLAG-IRTKS does not coimmunoprecipitate BirA∗ alone (*bottom row*) but is able to coimmunoprecipitate the indicated coexpressed FMNL2 derivatives tagged with BirA∗. IN; soluble cell lysate from transfected cells. E: eluate from FLAG beads. *G*, quantification of data shown in *A*–*C*. Percent of transfected cells with the indicated phenotypes. FMNL2.WT samples were analyzed in parallel, but images are not shown. *Black bars*: baseline filopodia formation, *yellow bars*: intermediate filopodia formation, and *red bars*: extensive dorsal and peripheral filopodia. N = 3, >100 cells/trial. Error bars represent SEM. *H*, quantification of data shown in *A*, *D*, and *E*. Percent of transfected cells with the indicated phenotypes. As in (*G*), FMNL2.WT samples were analyzed in parallel, but images are not shown. *Black bars*: baseline filopodia formation, *yellow bars*: intermediate filopodia formation, and *red bars*: extensive dorsal and peripheral filopodia. N = 3, >100 cells/trial. Error bars represent SEM. *I*, expression levels of the indicated mCherry-tagged FMNL2 derivatives relative to endogenous FMNL2 as detected by immunoblotting of whole cell lysates with anti-FMNL2 antibody. *Bottom panel*, relative expression levels of coexpressed FLAG-tagged IRTKS in the same lysates. FMNL2, formin-like 2.

The crystal structure of the FMNL2 N terminus suggests that docking of FMNL2 at the plasma membrane would induce membrane bending (12), and we observed previously that interference with the N-myristoylation signal in FMNL2 inhibited its ability to induce filopodia assembly (14). Similarly, IRTKS is thought to promote filopodia formation through its ability to induce negative membrane bends (26, 27, 35). To further explore the nature of the functional interaction between FMNL2 and IRTKS during filopodia assembly, we generated nonmyristoylated (G2A) (19) and actin-defective (I705A) (38) FMNL2 point mutant derivatives. Both these point mutant derivatives were highly deficient in their ability to induce filopodia assembly when expressed in A2058 cells despite being expressed at similar levels to the wildtype protein (Fig. 4, *B*, *D* and *G*–*I*). FMNL2.I705A-mCherry accumulates at the plasma membrane as expected (Fig. 4*B*), whereas FMNL2.G2A-mCherry is largely cytoplasmic (Fig. 4*D*). Surprisingly, coexpression of IRTKS is able to rescue filopodia assembly induced by FMNL2.I705A (Fig. 4, *C* and *G*) with abundant dorsal and peripheral filopodia present on the transfected cells. Coexpression of FMNL2.G2A with IRTKS does not induce filopodia assembly above the baseline induced by IRTKS alone (Fig. 4, *E* and *H*). Both FMNL2.I705A and FMNL2.G2A derivatives are coimmunoprecipitated by IRTKS suggesting that the failure of IRTKS to rescue FMNL2.G2A-induced filopodia assembly is not because the two proteins are unable to interact (Fig. 4*F*).

**Figure 5:**
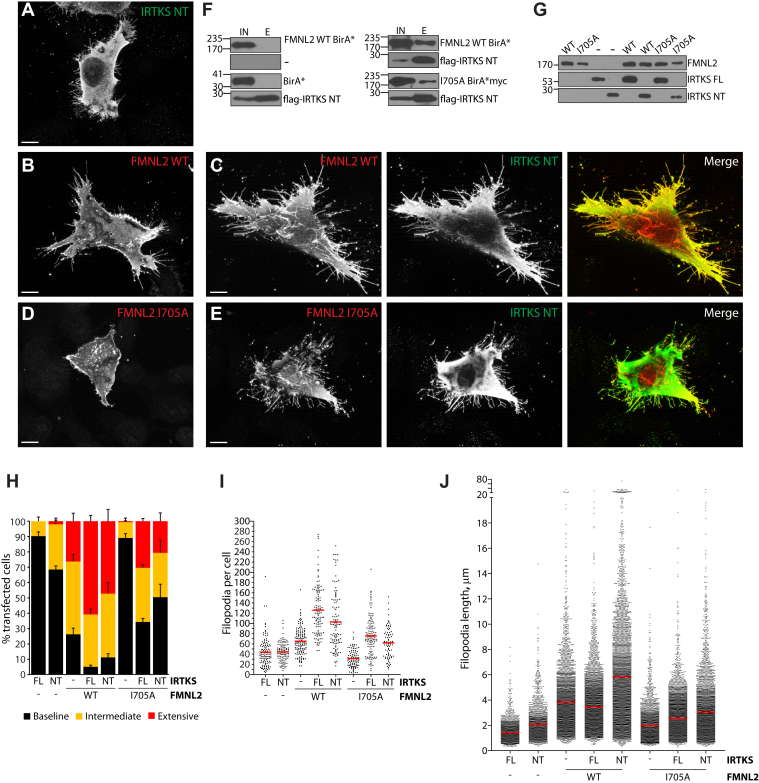
**The I-BAR domain of IRTKS is sufficient for the cooperative interaction with FMNL2.***A*, transient expression of IRTKS.NT induces filopodia assembly in A2058 cells. *B*, transient expression of FMNL2-mCherry induces filopodia formation in A2058 cells. *C*, coexpression of FLAG-IRTKS.NT (*green*) with FMNL2-mCherry (*red*) induces extensive formation of dorsal and peripheral filopodia. *D*, transient expression of FMNL2.I705A in A2058 cells does not induce filopodia formation. *E*, coexpression of IRTKS NT (*green*) with FMNL2.I705A (*red*) induces extensive formation of dorsal and peripheral filopodia. The scale bar represents 10 μm. *F*, *left panel*, FMNL2.BirA is not pulled down by FLAG affinity beads alone, and FLAG-IRTKS.NT does not coimmunoprecipitate BirA∗ alone. *Right panel*, FLAG-IRTKS.NT coimmunoprecipitates FMNL2.BirA∗ as well as FMNL2.I705A.BirA∗. *G*, relative expression levels of the indicated mCherry-tagged FMNL2 derivatives as detected by immunoblotting of whole cell lysates with anti-mCherry antibody. *Bottom panels*, relative expression levels of coexpressed FLAG-tagged IRTKS or IRTKS.NT in the same cell lysates. *H*, quantification of cell morphology data shown in (*A*–*E*). Percent of transfected cells with the indicated phenotypes. *Black bars*: baseline filopodia formation, *yellow bars*: intermediate filopodia formation, and *red bars*: extensive dorsal and peripheral filopodia. N = 3, >100 cells/trial. Error bars represent SEM. *I*, quantification of data shown in (*A*–*E*). The average number of filopodia present on transfected cells. N = 3, >30 cells/trial. Error bars represent SEM. *J*, quantification of data shown in (*A*–*E*). Length of individual filopodia in transfected cells. N = 3, individual filopodia measured on >30 cells/trial. *Red bar*: average length. E, eluate from FLAG beads; FMNL2, formin-like 2; I-BAR, Inverse-Bin-Amphiphysin-Rvs; IN, input: soluble cell lysate from transfected cells.

The I-BAR domain is the minimal membrane-binding unit and also able to bind and bundle F-actin (30). We therefore wanted to determine if the I-BAR domain of IRTKS is sufficient for its cooperative effects with FMNL2. As with expression of full-length IRTKS, expression of the isolated N terminus (IRTKS.NT) induced formation of short filopodia, albeit somewhat longer than those induced by full-length IRTKS (Fig. 5*J*). IRTKS.NT was also able to potentiate filopodia assembly induced by coexpressed FMNL2.WT (Fig. 5, *B*, *C* and *H*–*J*). We noted that the number of filopodia produced by cells coexpressing FMNL2 and full-length IRTKS was greater than the number of filopodia on cells coexpressing IRTKS.NT and FMNL2 (Fig. 5*I*). The filopodia produced in the IRTKS.NT + FMNL2 cells, however, were notably longer (Fig. 5*J*). The IRTKS.NT was also sufficient to coimmunoprecipitate FMNL2 (Fig. 5*F*) consistent with the interaction of FMNL2 with IRTKS being required for their cooperative effects on filopodia formation. Together, these observations confirm that the isolated I-BAR domain of IRTKS is sufficient for its cooperative effects on filopodia assembly with FMNL2.

We next assessed the effects of coexpression of IRTKS.NT with the actin-deficient FMNL2.I705A derivative. As with FMNL2.WT, IRTKS.NT is sufficient to coimmunoprecipitate FMNL2.I705A (Fig. 5*F*). Coexpression of IRTKS.NT with FMNL2.I705A had very similar effects to coexpression of I705A with full-length IRTKS. More cells produce extensive filopodia (Fig. 5, *E*, *H* and *I*), and the filopodia are somewhat longer than those produced by coexpression of I705A with full-length IRTKS. This effect, however, is markedly diminished in comparison to coexpression of IRTKS.NT with FMNL2.WT suggesting that C-terminal regions of IRTKS are required for synergy with FMNL2 in the absence of FH2 function.

**Figure 6:**
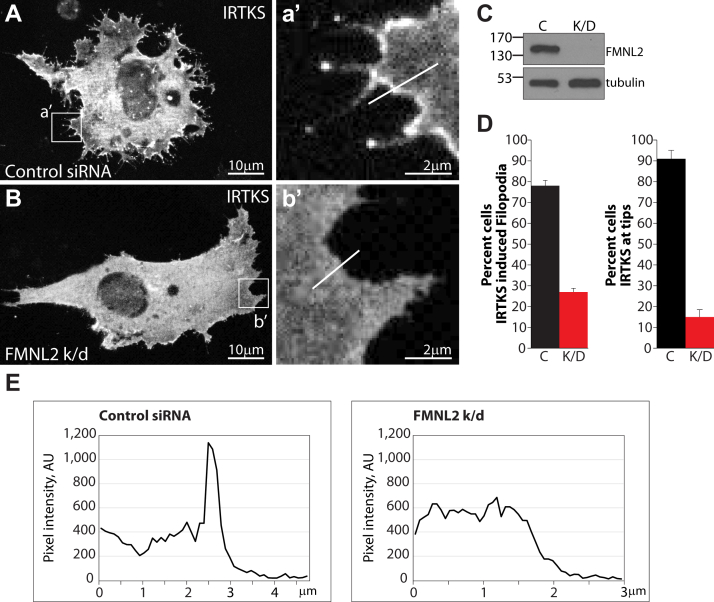
**IRTKS-induced filopodia assembly is FMNL2 dependent.***A*, IRTKS-GFP localizes to the edge of the plasma membrane and induces the formation of short filopodia in A2058 cells transfected with a control siRNA duplex. *a'*, IRTKS-GFP localizes to filopodia tips and the plasma membrane. *White line* was used to generate the pixel intensity plot show in (*E*). *B*, FMNL2 depletion inhibits filopodia assembly by IRTKS-GFP. The scale bar represents 10 μm. *b'*, IRTKS-GFP does not localize to the edge of the plasma membrane and fails to induce filopodia in FMNL2 knockdown (k/d) A2058 cells. *White line* was used to generate the pixel intensity plot show in (*E*). *C*, immunoblot confirming extent of FMNL2 depletion in siRNA transfected cells. Tubulin was used as a loading control. *D*, quantification of data shown in (*A* and *B*). The *left chart* indicates percent of transfected cells with IRTKS-GFP-induced filopodia assembly in control (c) and FMNL2-depleted cells (k/d). *Right chart* indicates percent of transfected cells with IRTKS-GFP at the tips of filopodia in control (c) and FMNL2-depleted cells (k/d). N = 3, >100 cells/trial. Error bars represent SEM. *E*, pixel intensity plots from lines shown in (*a'*) and (*b'*) indicating a clear accumulation of IRTKS-GFP at the edge of control cells but not FMNL2-depleted cells. Similar results were obtained with a second siRNA duplex targeting FMNL2 (Fig. S4) and with FMNL2 k/d in A375 cells (Fig. S5). *C*, whole cell lysates from control siRNA transfected cells; FMNL2, formin-like 2; K/D, whole cell lysates from cells transfected with siRNA targeting.

siRNA-mediated knockdown of IRTKS inhibited FMNL2-dependent filopodia assembly (Fig. 3). To determine if the reverse is true, we tested the effects of FMNL2 knockdown on targeting of IRTKS to the plasma membrane and IRTKS-induced filopodia assembly (Fig. 6). In control cells, IRTKS-GFP accumulates at the cell periphery and induces filigreed cellular protrusions and short filopodia with IRTKS clearly localized at their tip (Fig. 6, A, a', *D* and *E*). In FMNL2 knockdown cells, IRTKS no longer localizes to the plasma membrane and no longer induces filopodia formation (Fig. 6, B, b', *D* and *E*). Similar results were obtained in A375 cells and when a second siRNA duplex was used to knockdown FMNL2 expression in A2058 cells (Figs. S4 and S5). Together, our results suggest that FMNL2 and IRTKS are mutually dependent cofactors required for filopodia assembly.

## Discussion

FMNL2 and FMNL3 are paralogs associated with filopodia assembly in a variety of cell types (3, 13). The two proteins share significant sequence homology, and both proteins are targeted to the plasma membrane by N-myristoylation. Despite their similarity, they exhibit distinct subcellular localizations within the filopodia. We find that FMNL3 localizes to the filopodia tip, consistent with current models of filopodia assembly where formins function as part of the actin polymerization machinery at the tip complex (4). In contrast, FMNL2 distributes along the length of the filopodia, consistent with a distinct function in this process. We identified the membrane-binding I-BAR proteins IRTKS and IRSp53 as FMNL2-binding proteins and show that they act cooperatively with FMNL2 to induce filopodia assembly in two human melanoma cell lines. However, in these cells, only IRTKS is required for FMNL2 function, whereas IRSp53 is not. It is not clear why this should be the case given the similarities between the two proteins, and it is possible that IRSp53 may provide a similar essential function for FMNL2 in other cell types. Nevertheless, our results show a mutual codependency of IRTKS and FMNL2 in our assays. FMNL2 depletion inhibits IRTKS concentration at the periphery of the plasma membrane and inhibits IRTKS-induced assembly of short filopodia. To our knowledge, the FMNL2 dependence of IRTKS recruitment to the plasma membrane is the first time such an interaction has been reported for an I-BAR protein. In turn, IRTKS depletion inhibits FMNL2-induced filopodia assembly but does not affect targeting of FMNL2 to the plasma membrane. Surprisingly, IRTKS coexpression can rescue filopodia assembly induced by the I705A actin-binding mutant of FMNL2 but not the myristoylation-defective G2A mutant. This suggests that targeting of FMNL2 to the plasma membrane plays a critical role in the initiation of filopodia assembly and that FMNL2 plays a novel role in this process.

### IRTKS, IRSp53, FMNL2, and cell morphology

Coexpression of FMNL2 with IRTKS induced a striking increase in the number of cells with extensive dorsal and peripheral filopodia. These structures were notably distinct from the filopodia induced by coexpression of IRSp53 with FMNL2, which were longer and generally more restricted to the cell periphery and less so to the dorsal surface. This is consistent with previous reports where IRTKS generally induces shorter filopodia than IRSp53 (30). IRTKS is also more associated with formation of dorsal filopodia and ruffles as well as apical microvilli in intestinal epithelium (27, 29). The distinct phenotypes induced by IRTKS alone *versus* IRTKS coexpressed with FMNL2 are reminiscent of previous scanning electron microscopy studies of melanoma cell morphology (39). These found that subconfluent cultures of A375 melanoma cells exhibited two distinct cellular phenotypes. In the first, cells are flatter with numerous short microvilli on their dorsal surface, very similar to the phenotype we see in IRTKS-expressing cells. In the second, cells are thicker with extensive dorsal ruffles and longer filopodia at the periphery and dorsal surface, very similar to the thicker “extensive” filopodia phenotype we see in FMNL2–IRTKS-expressing cells. We propose it is likely that relative levels of IRTKS and FMNL2 activity may be responsible for the partitioning of these cells between these two cellular morphologies. The function of the distinct dorsal structures seen on “thick” melanoma cells is not clear, but it was initially suggested that they may be involved in phagocytosis (39). Given the connection between FMNL2 and melanoma metastasis (31) as well as the association of filopodia with tumor cell invasion (40), it is tempting to speculate that the phenotypic shift induced by the cooperative effects of FMNL2 and IRTKS on cell morphology could be connected to the proinvasive function of FMNL2 (14, 41).

### FMNL2, IRTKS, and filopodia assembly

IRTKS, and other I-BAR proteins, are able to induce negative membrane bends that are thought to initiate filopodia assembly by the release of plasma membrane tension (6, 27, 35). A similar function was proposed for FMNL2 based on the modeled docking of its N terminus at the plasma membrane (12). Our results support this model, where targeting of FMNL2 to the plasma membrane is critical for FMNL2-dependent filopodia assembly. First, we find that the G2A mutation blocks FMNL2-induced filopodia assembly despite this derivative retaining an intact FH2 domain. Second, we find FMNL2-induced filopodia assembly is dependent on IRTKS activity and that the minimal membrane-binding domain of IRTKS is sufficient for cooperative induction of filopodia assembly when coexpressed with FMNL2. IRTKS-induced filopodia are also FMNL2 dependent. Third, we find that expression of IRTKS is sufficient to rescue filopodia assembly induced by FMNL2.I705A suggesting that the ability of FMNL2 to regulate actin dynamics is not absolutely essential in this process. Indeed, coexpression of IRTKS with FMNL2.I705A essentially restores the effects equivalent to the wildtype protein. How does IRTKS rescue the I705A mutant? The simplest model is that the F-actin binding and bundling properties of IRTKS are sufficient to substitute for the function of the FMNL2 FH2 domain during filopodia assembly. We do note that the effects of IRTKS.NT are diminished when coexpressed with FMNL2.I705A as opposed to FMNL2.WT. This suggests that in addition to the I-BAR domain, a second F-actin binding motif is required that can be provided either by FH2 or by the C-terminal domain of IRTKS. Alternatively, we cannot rule out that a putative FMNL2–IRTKS complex recruits additional actin regulatory factors that drive filopodia assembly dependent upon the SH3 domain or WH2-like motif of IRTKS. This would be consistent with recent reports that specific cytoskeletal regulatory proteins are not required for filopodia assembly *in vitro* as long as the requisite F-actin binding, bundling, and anticapping activities are provided (7).

**Figure 7:**
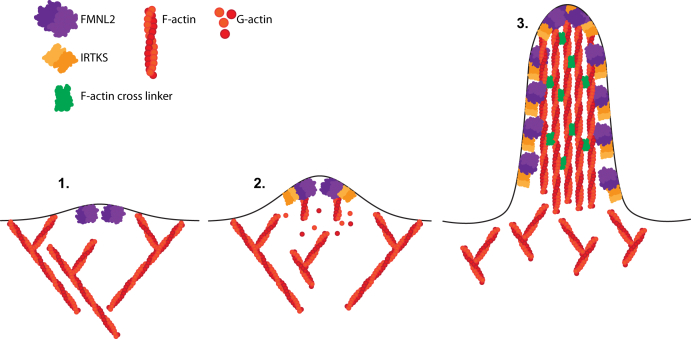
**Cooperative assembly of filopodia by FMNL2 and IRTKS.** In our model, filopodia assembly begins with (1) docking of FMNL2 at the plasma membrane. (2) IRTKS is then recruited by FMNL2-induced membrane bending and is maintained at the membrane by its interaction with FMNL2. Continued membrane bending by the FMNL2–IRTKS complex releases membrane tension to facilitate actin polymerization and initiate filopodia assembly. (3) F-actin may be bundled by FMNL2 and IRTKS to promote continued filopodia growth that is maintained both by on-going FMNL2–IRTKS-induced membrane tubulation and by the anticapping activity of FMNL2. FMNL2, formin-like 2.

We feel it is unlikely that actin binding is the sole role of IRTKS in its cooperative association with FMNL2. In IRTKS knockdown cells, FMNL2 is still targeted to the plasma membrane and the wildtype FMNL2 should still be able to regulate actin assembly. Nonetheless, the depletion of IRTKS blocks FMNL2-induced filopodia formation. Conversely, FMNL2 knockdown inhibits accumulation of IRTKS at the periphery of the plasma membrane. These results suggest a hierarchical relationship in which FMNL2 accumulates at the plasma membrane on its own where it subsequently recruits IRTKS *via* FMNL2-induced membrane bending as well as through the FMNL2–IRTKS protein–protein interaction. Thus, we propose an updated model for FMNL2-induced filopodia assembly (Fig. 7). First, FMNL2 is targeted to the plasma membrane by N-myristoylation where it initiates membrane bending. Next, IRTKS is recruited to sites of FMNL2-induced membrane bending, and its localization at the membrane is stabilized by a direct FMNL2–IRTKS interaction. The two proteins then nucleate the formation of a nascent tip complex that promotes actin polymerization to initiate filopodia assembly. Finally, filopodia growth is sustained by continued membrane association of FMNL2 and IRTKS as well as by anticapping and F-actin bundling activities that can be provided in part by the FH2 of FMNL2 as well as by the I-BAR domain of IRTKS. Our model neither does rule out the recruitment of additional actin remodeling proteins by FMNL2 or IRTKS nor does exclude an anticapping role for FMNL2 at the tip complex, although this does not appear to be essential with higher levels of IRTKS expression. Given the concordance of our results in both A375 and A2058 cell lines, we feel that our model for FMNL2–IRTKS cooperation may be generally applicable in human melanoma cells.

## Experimental procedures

### Reagents and plasmids

Subcloning of full-length human FMNL2 complementary DNA into pEF-mCherry was previously described (14, 16). FMNL2 was subcloned into pBirA∗-N1 (a gift from Laura Trinkle-Mulcahy) using standard techniques. pECE-M2-BAIAP2 wt was a gift from Anne Brunet (Addgene plasmid #31656; http://n2t.net/addgene:31656; Research Resource Identifier [RRID]: Addgene_31656). Gap43-mCherry (mCherry-Mem) was a gift from Catherine Berlot (Addgene plasmid #55779; http://n2t.net/addgene:55779; RRID: Addgene_55779). mEmerald-MYR-N-5 was a gift from Michael Davidson (Addgene plasmid # 54198; http://n2t.net/addgene:54198; RRID: Addgene_54198). The full-length IRTKS (BAIAP2L1) complementary DNA was generated from A375 mRNA as described (14) using the following oligos 5′B2L1.Bam CCCGGATCCATGTCCCGGGGGCC; 3′B2L1.Spe1 CCCCACTAGTTCATCGAATGATGGGTGCCGAGC and subcloned into pEF-FLAG, and into pEGFP-N1 using 5′Bgl.2L1 CCCCAGATCTATGTCCCGGGGGCC and 3′Sal.2L1 CCTCGTCGACGCTCCTCGAATGATGGGTG. The following antibodies were used in this study: chicken anti-FMNL2 (14), mouse anti-BAIAP2L1 (SCBT; catalog no.: sc-393838), mouse anti-IRSp53 (SCBT; catalog no.: sc-136470), mouse anti-α-tubulin (Sigma; catalog no.: T5168), mouse anti-FLAG (Sigma; catalog no.: F7425), rat 5f8 anti-RFP antibody (Chromatek), Donkey antimouse 488, Donkey anti-rabbit 488, Donkey antimouse 594, Donkey anti-rabbit 594 (Jackson Labs), and anti-FLAG-horseradish peroxidase (Sigma; catalog no.: A8592). Alexa Fluor 488 Phalloidin (Molecular Probes; catalog no.: A12379), Streptavidin agarose (Solulink; catalog no.: N1000-005), RFP-trap beads (Chromatek), and Anti-DYKDDDK affinity resin (GenScript; catalog no.: L00432).

### Cell culture, transfections, and treatments

A2058 (CRL-11147) and A375 (CRL-1619) melanoma cells were obtained from the American Type Culture Collection and cultured in Dulbecco's modified Eagle's medium (Wisent) supplemented with 10% fetal bovine serum (American Type Culture Collection) in 5% CO_2_ according to the supplied guidelines. Mycoplasma contamination was tested biweekly. Transient transfections were performed using polyethyleneimine (PEI) as described previously (14). Briefly, 1.5 μg total plasmid DNA was diluted in 50 μl Opti-MEM, 5 μl of 1 mg/ml PEI was added, and the mixture was incubated for 25 to 30 min at room temperature. The DNA–PEI mix was added to cells in 1 ml of Opti-MEM and left for 5 h under normal culture conditions. At the end of 5 h, the media were replaced with 2 ml of the appropriate culture medium. siRNA-mediated knockdown was performed as previously described (42) using Dharmafect1 (PerkinElmer) and the following siRNA duplexes: FMNL2 siRNA Duplex1 (IDT; hs.Ri.FMNL2.13.1); FMNL2 siRNA duplex2 (IDThs.Ri.FMNL2.13.2); IRTKS duplex1 (IDT, hs.Ri.BAIAP2L1.13.1); IRTKS duplex2 (IDT, hs.Ri.BAIAP2L1.13.2); and IRSp53 duplex1 (IDT, hs.Ri.BAIAP2.13.1).

### BioID screen

We used a metabolic labeling (stable isotope labeling by amino acids in cell culture)–based quantitative BioID approach to map the FMNL2 interactome. Proteins were filtered *in silico* for known background contaminants (43) and prioritized based on gene function as previously described (42). Full results of the screen are to be reported elsewhere (Fox *et al.*, unpublished results). Candidate interactors were first tested for the ability of FMNL2-BirA to biotinylate the endogenous protein when transiently expressed in HEK293T/17 cells and then confirmed by co-IP of epitope-tagged derivatives of the proteins of interest with FMNL2-mCherry or FMNL2-BirA∗. Co-IPs were performed as previously described (44). Briefly, transfected cells were scraped from their dish, washed three times in 1× PBS, and lysed on ice for 20 min in co-IP buffer (50 mM Tris [pH 7.0], 150 mM NaCl, 1 mM EDTA, 5 mM NaF, 0.5% Triton X-100, and protease inhibitors). Lysates were cleared by centrifugation (10 min, 16,000*g*), and the supernatant was incubated with anti-DYKDDDDK agarose beads or RFP-Trap beads (ChromoTek, rta) for 2 h at 4 °C. The beads were washed three times in co-IP buffer, and the bound proteins eluted in 1× Laemmli loading buffer. Bound proteins were detected by immunoblotting for their epitope tags.

### Immunofluorescence

Cells were prepared for immunofluorescence as described previously (42). Briefly, cells cultured on acid-washed glass coverslips were fixed for 10 min directly in 4% paraformaldehyde freshly prepared in PHEM (Pipes, Hepes, EGTA, and MgCl_2_) buffer (45). Following fixation, the cells were permeabilized and blocked for 20 min in 0.3% Triton X-100, 5% donkey serum in 1× PBS. The coverslips were washed in 1× PBS and incubated with the appropriate primary antibody in 0.03% Triton X-100 and 5% donkey serum in 1× PBS for 1 h at room temperature. The coverslips were washed three times in 1× PBS and then incubated with secondary antibody in the same solution for 1 h at room temperature. After washing in 1× PBS, the coverslips were rinsed in double-distilled water, and mounted in Vectashield with 4′,6-diamidino-2-phenylindole and sealed with nail polish.

### Microscopy

All microscopy was performed on a Zeiss AXIO Imager.Z1 with a Zeiss Apotome.2 structured illumination system for optical sectioning using a 63× (numerical aperture of 1.4) oil immersion lens and a Zeiss AxioCam HRm camera (60N-C 1” 1.0X 426114) controlled with AxioVision (Zeiss, release 4.8.2). Coverslips were mounted in Vectashield (Vector Labs) with or without 4′,6-diamidino-2-phenylindole. Figures were prepared in Adobe Photoshop and Adobe Illustrator. Cell morphology in terms of dorsal ruffles and dorsal and peripheral filopodia was assessed visually and divided into three categories: “baseline,” “intermediate,” and “extensive” based on filopodia length, number, and cell morphology. Peripheral filopodia lengths were measured using ImageJ (NIH). Cell height was measured manually based on the difference between the first and last focal planes for each cell.

## Data availability

All data are included in the article.

## Supporting information

This article contains supporting information.

## Conflict of interest

The authors declare that they have no conflicts of interest with the contents of this article.
