# Supplementary material for: Cooperative assembly of filopodia by the formin FMNL2 and I-BAR domain protein IRTKS
Source: J Biol Chem. 2022 Sep 19;298(11):102512. doi: 10.1016/j.jbc.2022.102512 (PMC9579038; doi:10.1016/j.jbc.2022.102512)
Supplement: Supplemental Figures S1–S5 Legends [file mmc1.docx]

**Supporting Information**

**Figure S1. Classification of cell morphology.** Transiently transfected A2058 and A375 cells were classified visually into 3 categories based on cell morphology, filopodia formation and filopodia distribution. **Baseline**, typified by the presence of few short filopodia at the cell periphery and no dorsal filopodia or ruffles**. Intermediate,** typified by the presence of longer peripheral filopodia but few dorsal structures. **Extensive,** typified by extensive formation of longer peripheral and dorsal filopodia and dorsal ruffles. Scale bar=10μm.

**Figure S2. Synergistic activation of filopodia assembly by FMNL2 and IRTKS in A375 cells. A)** Gap43-mCherry expression in A375 cells does not induce filopodia formation. **B)** Expression of Flag-IRTKS induces assembly of moderate amounts of short filopodia. **C)** FMNL2-mCherry expression induces filopodia formation. **D)** Co-expression of Flag-IRTKS with FMNL2-mCherry induces extensive formation of dorsal and peripheral filopodia. Scale bar=10μm. **E)** Quantification of data shown in A-D. Percent of transfected cells with the indicated phenotypes. black bars: baseline filopodia formation, yellow bars: intermediate filopodia formation, red bars: extensive dorsal and peripheral filopodia. N=3, >100 cells/trial. Error bars=SEM. **F)** As in (A), Gap43 mCherry expression in A375 cells does not induce filopodia formation. **G)** Expression of myc-IRSp53 has modest effects on the formation of peripheral filopodia. **H)** FMNL2-mCherry expression induces filopodia formation. **I)** Co-expression of myc-IRSp53 with FMNL2-mCherry induces extensive formation of long peripheral filopodia. Scale bar=10μm. **J)** Quantification of data shown in F-I. Percent of transfected cells with the indicated phenotypes. black bars: baseline filopodia formation, yellow bars: intermediate filopodia formation, red bars: extensive dorsal or peripheral filopodia. N=3, >100 cells/trial. Error bars=SEM.

**Figure S3. FMNL2-induced filopodia assembly is IRTKS dependent in A2058 and A375 cells. A,B)** Gap43 mCherry expression does not affect filopodia formation in A2058 cells transfected with a control siRNA duplex or in IRTKS knockdown (k/d) cells using a second siRNA duplex. **C)** FMNL2-mCherry expression induces filopodia formation in control siRNA transfected cells. **D)** FMNL2-mCherry is still targeted to the plasma membrane in IRTKS depleted cells, but filopodia formation is inhibited. Scale bar=10μm. **E)** FMNL2-mCherry expression in A2058 induces an increase in cell height that is inhibited by siRNA-mediated knockdown of IRTKS expression. N=3, >50 cells/trial, red bars: average height. **F)** Immunoblots confirming extent of IRTKS depletion in siRNA transfected cells. “-”: whole cell lysates from untransfected cells, C: whole cell lysates from control siRNA transfected cells. K/D: whole cell lysates from cells transfected with siRNA targeting IRTKS. Tubulin was used as a loading control. **G,H)** Gap43 mCherry expression does not affect filopodia formation in A375 cells transfected with a control siRNA duplex or in IRTKS knockdown (k/d) cells. **I)** FMNL2-mCherry expression induces filopodia formation in control siRNA transfected cells. **J)** FMNL2-mCherry is still targeted to the plasma membrane in IRTKS depleted cells, but filopodia formation is inhibited. Scale bar=10μm. **K)** FMNL2-mCherry expression in A375 induces an increase in cell height that is inhibited by siRNA-mediated knockdown of IRTKS expression. N=3, >50 cells/trial, red bars: average height. **L)** Immunoblots confirming extent of IRTKS depletion in siRNA transfected cells. “-”: whole cell lysates from untransfected cells, C: whole cell lysates from control siRNA transfected cells. K/D: whole cell lysates from cells transfected with siRNA targeting IRTKS. Tubulin was used as a loading control.

**Figure S4. IRTKS induced filopodia assembly is FMNL2 dependent (2^nd^ siRNA). A)** IRTKS-GFP localizes to the edge of the plasma membrane and induces the formation of short filopodia in A2058 cells transfected with a control siRNA duplex. **a’)** IRTKS-GFP localizes to filopodia tips and the plasma membrane. **B)** FMNL2 depletion using a second siRNA duplex inhibits filopodia assembly by IRTKS-GFP. Scale bar=10μm. **b’)** IRTKS-GFP does not localize to the edge of the plasma membrane and fails to induce filopodia in FMNL2 knockdown A2058 cells. **C)** Immunoblot confirming extent of FMNL2 depletion in siRNA transfected cells, C: whole cell lysates from control siRNA transfected cells. K/D: whole cell lysates from cells transfected with siRNA targeting FMNL2. Tubulin was used as a loading control. **D)** Quantification of data shown in (A,B). The left chart indicates percent of transfected cells with IRTKS-GFP induced filopodia assembly in control (c) and FMNL2 depleted cells (k/d). Right chart indicates percent of transfected cells with IRTKS-GFP at the tips of filopodia in control (c) and FMNL2 depleted cells (k/d).

**Figure S5. IRTKS activity is FMNL2 dependent in A375 cells. A)** IRTKS-GFP localizes to the edge of the plasma membrane and induces the formation of short filopodia in A375 cells transfected with a control siRNA duplex. **a’)** IRTKS-GFP localizes to filopodia tips and the plasma membrane. **B)** FMNL2 depletion inhibits filopodia assembly by IRTKS-GFP. Scale bar=10μm. **b’)** IRTKS-GFP does not localize to the edge of the plasma membrane and fails to induce filopodia in FMNL2 knockdown A375 cells. **C)** Immunoblot confirming extent of FMNL2 depletion in siRNA transfected cells, C: whole cell lysates from control siRNA transfected cells. K/D: whole cell lysates from cells transfected with siRNA targeting FMNL2. Tubulin was used as a loading control. **D)** Quantification of data shown in (A,B). The left chart indicates percent of transfected cells with IRTKS-GFP induced filopodia assembly in control (c) and FMNL2 depleted cells (k/d). Right chart indicates percent of transfected cells with IRTKS-GFP at the tips of filopodia in control (c) and FMNL2 depleted cells (k/d). **E)** IRTKS expression in A375 cells induces an increase in cell height that is inhibited by siRNA-mediated knockdown of FMNL2 expression. N=3, >50 cells/trial, red bars: average height. **F)** IRTKS expression in A2058 cells induces an increase in cell height that is inhibited by siRNA-mediated knockdown of FMNL2 expression. N=3, >50 cells/trial, red bars: average height.
