# Supplementary figures and images for: Cooperative assembly of filopodia by the formin FMNL2 and I-BAR domain protein IRTKS
Source: J Biol Chem. 2022 Sep 19;298(11):102512. doi: 10.1016/j.jbc.2022.102512 (PMC9579038; doi:10.1016/j.jbc.2022.102512)

**Figure S1**

**Baseline**

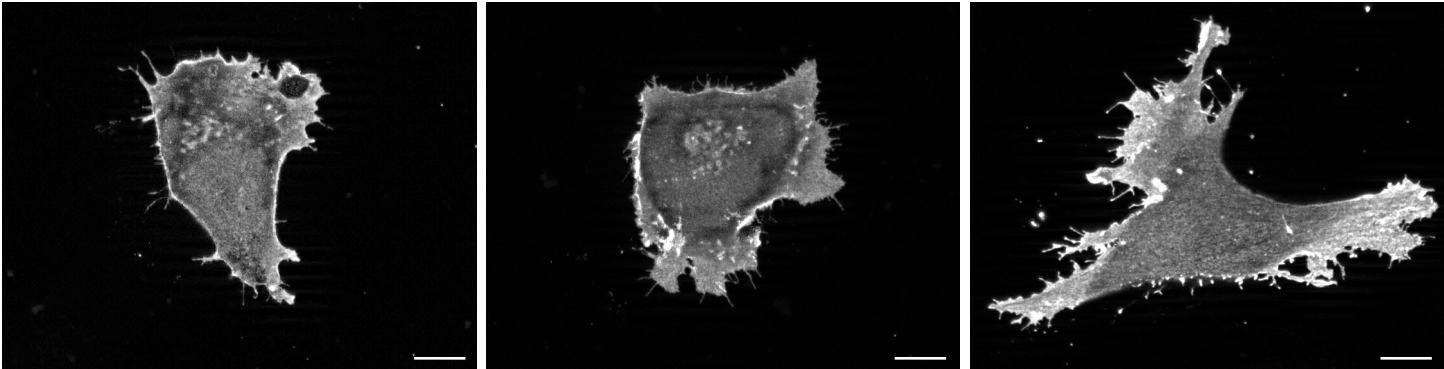

**Intermediate**

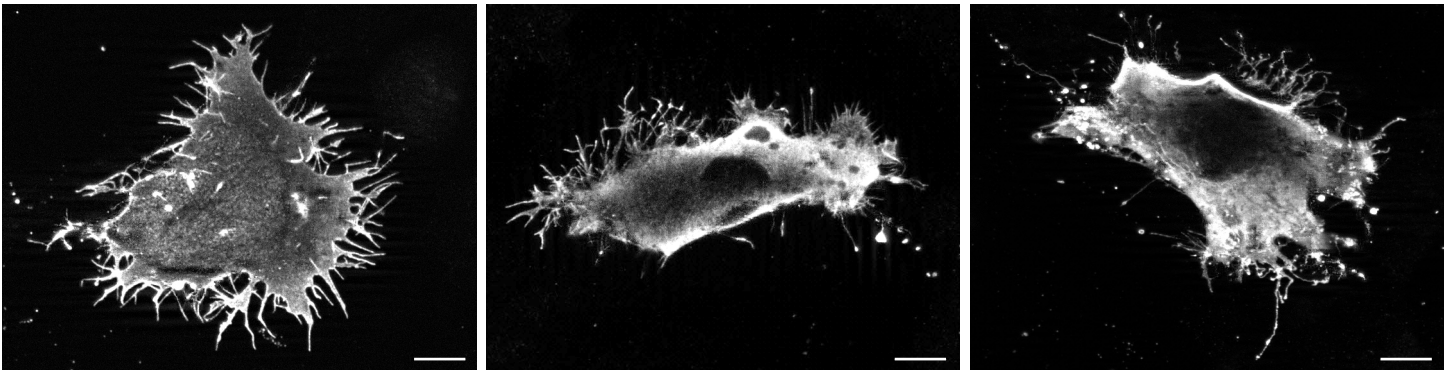

**Extensive**

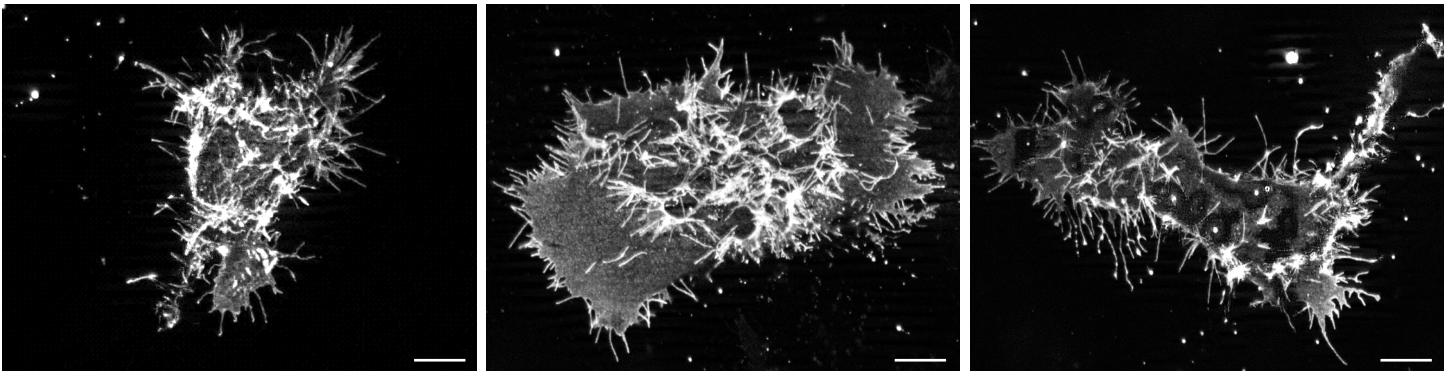

Supplement: Supplemental Figure S1 [file mmc2.pdf]

Figure S2

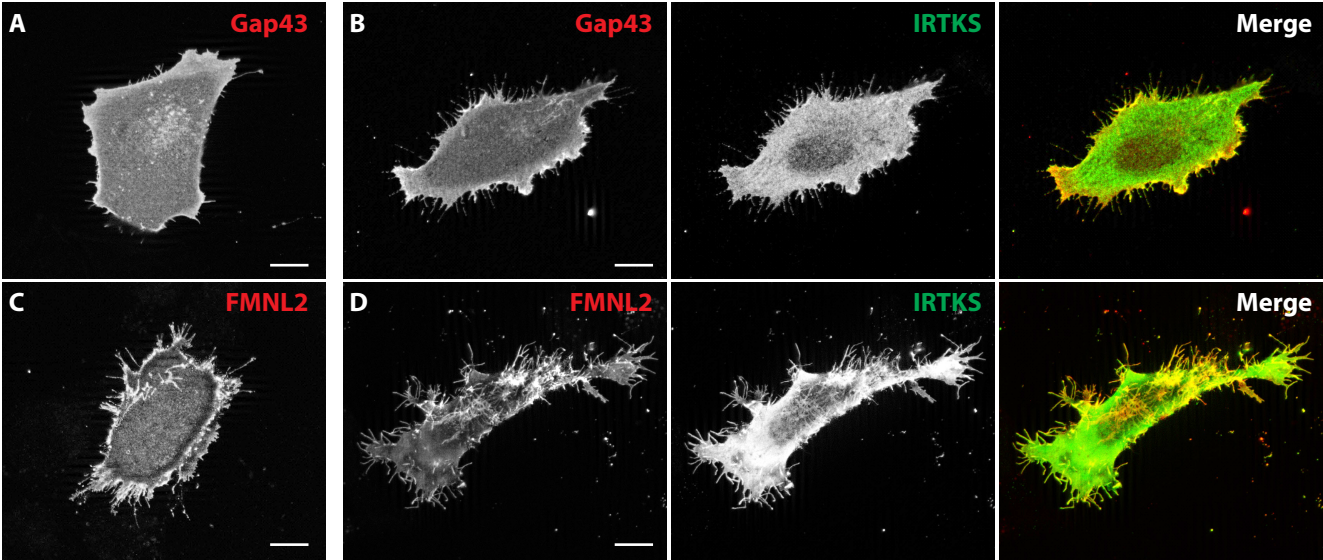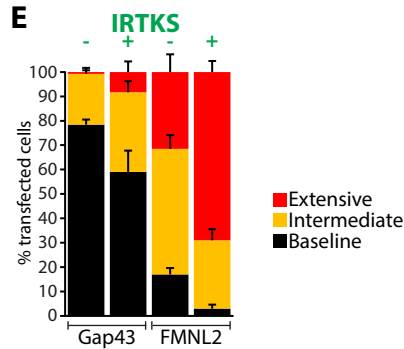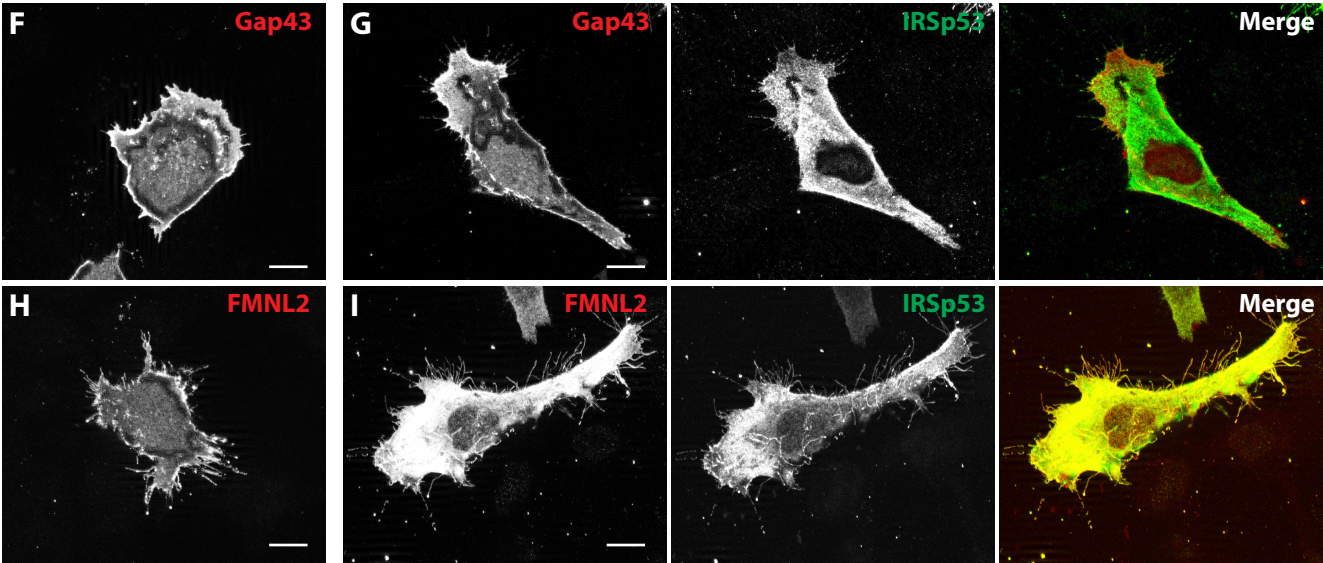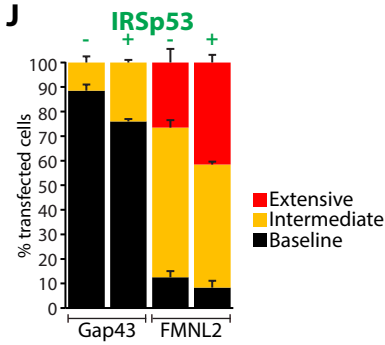

Supplement: Supplemental Figure S2 [file mmc3.pdf]

Figure S3

A2058 IRTKS k/d duplex 2

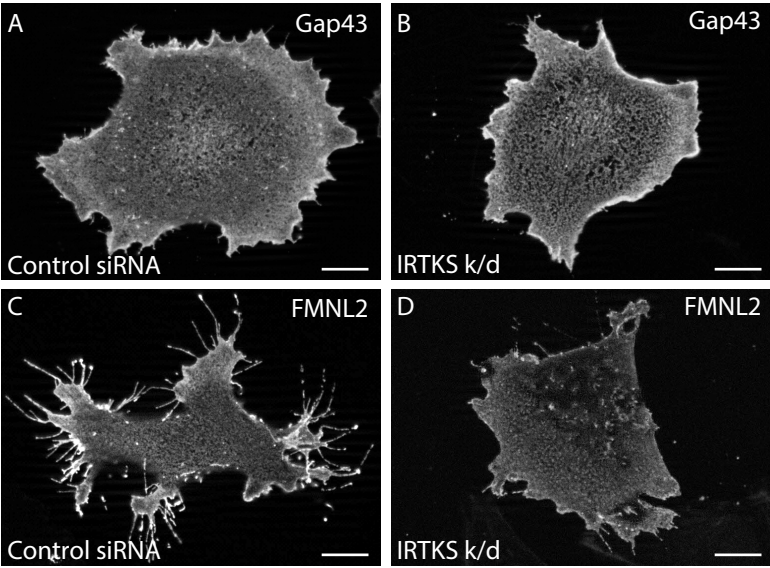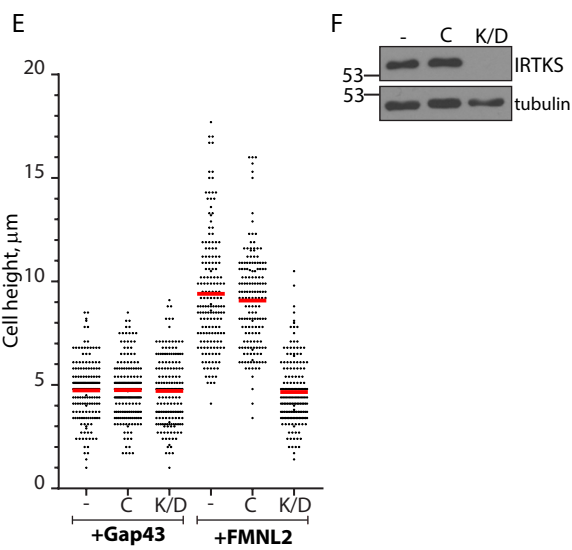

A375 IRTKS k/d

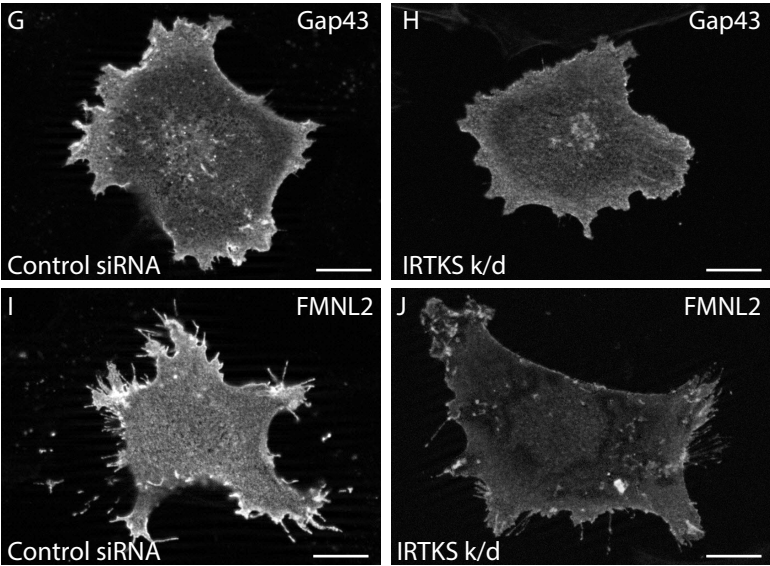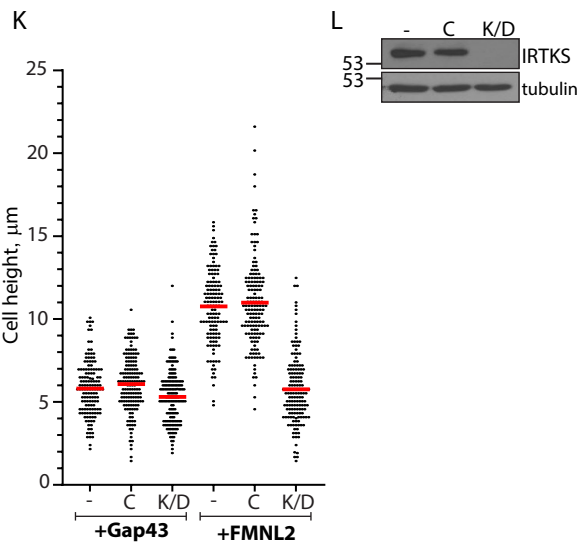

Supplement: Supplemental Figure S3 [file mmc4.pdf]

Figure S4

A2058 FMNL2 Duplex 2

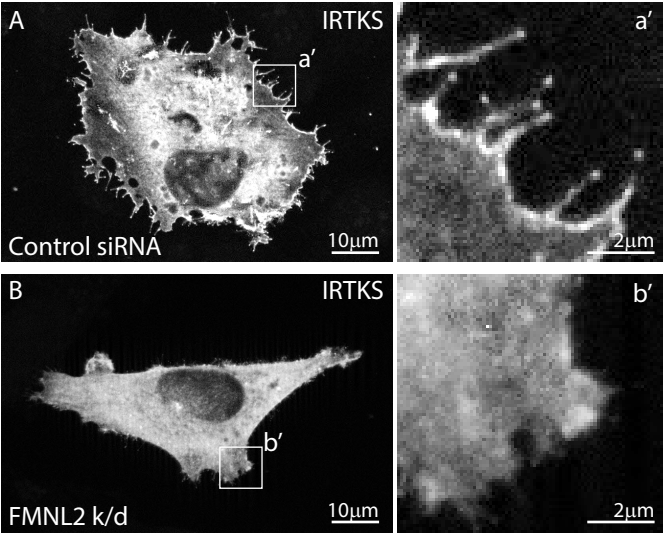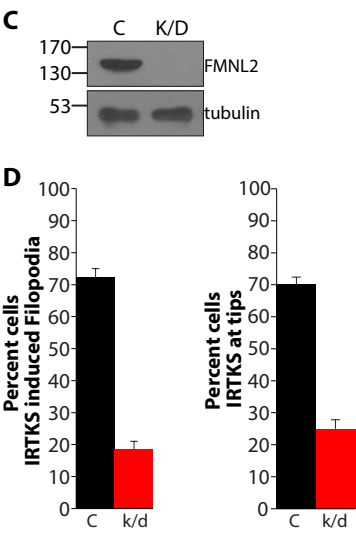

Supplement: Supplemental Figure S4 [file mmc5.pdf]

Figure S5

A375 FMNL2 K/D

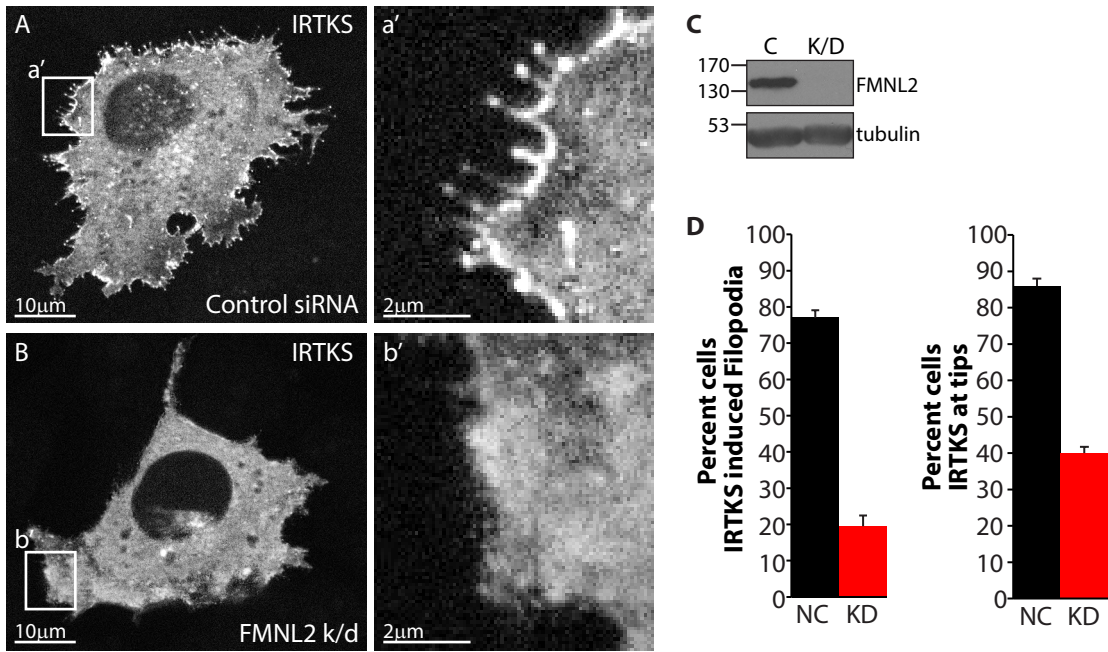

**E** A375 FMNL2 KD IRTKS o/e

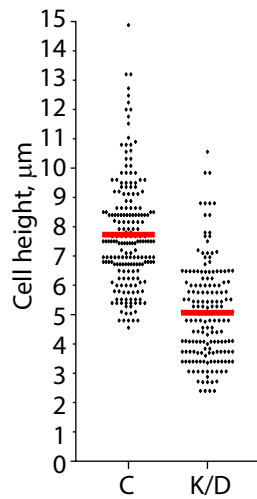

**F** A2058 FMNL2 KD IRTKS o/e

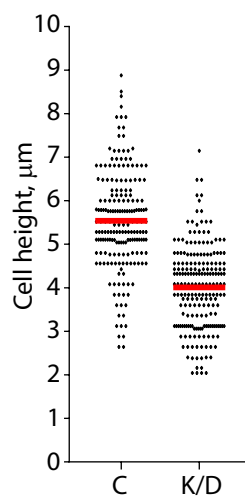

Supplement: Supplemental Figure S5 [file mmc6.pdf]
